# Identification of *Lasiodiplodia pseudotheobromae* Causing Fruit Rot of Citrus in China

**DOI:** 10.3390/plants10020202

**Published:** 2021-01-21

**Authors:** Jianghua Chen, Zihang Zhu, Yanping Fu, Jiasen Cheng, Jiatao Xie, Yang Lin

**Affiliations:** 1 Hubei Key Laboratory of Plant Pathology, Huazhong Agricultural University, Wuhan 430070, China; jianghuachen@webmail.hzau.edu.cn (J.C.); Zhuzihang@webmail.hzau.edu.cn (Z.Z.); yanpingfu@mail.hzau.edu.cn (Y.F.); jiasencheng@mail.hzau.edu.cn (J.C.); jiataoxie@mail.hzau.edu.cn (J.X.); 2 National R & D Center for Citrus Postharvest Technology, Huazhong Agricultural University, Wuhan 430070, China

**Keywords:** citrus, fruit decay, *Lasiodiplodia pseudotheobromae*

## Abstract

Considering the huge economic loss caused by postharvest diseases, the identification and prevention of citrus postharvest diseases is vital to the citrus industry. In 2018, 16 decayed citrus fruit from four citrus varieties—Satsuma mandarin (*Citrus unshiu*), Ponkan (*Citrus reticulata* Blanco cv. Ponkan), Nanfeng mandarin (*Citrus reticulata* cv. nanfengmiju), and Sugar orange (*Citrus reticulata* Blanco)—showing soft rot and sogginess on their surfaces and covered with white mycelia were collected from storage rooms in seven provinces. The pathogens were isolated and the pathogenicity of the isolates was tested. The fungal strains were identified as *Lasiodiplodia pseudotheobromae* based on their morphological characteristics and phylogenetic analyses using the internal transcribed spacer regions (ITS), translation elongation factor 1-α gene *(TEF*), and beta-tubulin (*TUB*) gene sequences. The strains could infect wounded citrus fruit and cause decay within two days post inoculation, but could not infect unwounded fruit. To our knowledge, this is the first report of citrus fruit decay caused by *L. pseudotheobromae* in China.

## 1. Introduction

Citrus is the second-largest fruit crop in China. It is cultivated in over 20 provinces, with an area of approximately 2.5 million ha [1,2]. China has an over 4000-year history of citrus cultivation. There are an abundant and excellent variety of resources in China. Fruit decay is a common disease during postharvest transportation and storage. Generally, the decay rate is 20% to 30%, and sometimes it can reach up to 50%, which causes serious postharvest economic losses [3]. Therefore, disease control is vital for the postharvest period of citrus fruit.

Common postharvest citrus diseases include green mold, blue mold, sour rot, anthracnose, Diplodia stem-end rot, Diaporthe stem-end rot, brown rot, and Alternaria stem-end rot, etc. In 2018, a survey of postharvest citrus diseases in storage rooms found that the average fruit decay rate exceeded 30% in seven provinces. In addition to the common postharvest diseases, 16 decayed fruit showing soft rot and soggy lesions with dense white hyphae were also observed. In this study, the causal agent of the citrus fruit rot disease was determined via morphological observation, molecular identification, and pathogenicity tests.

## 2. Results

### 2.1. Fungal Isolates

**Figure 1:**
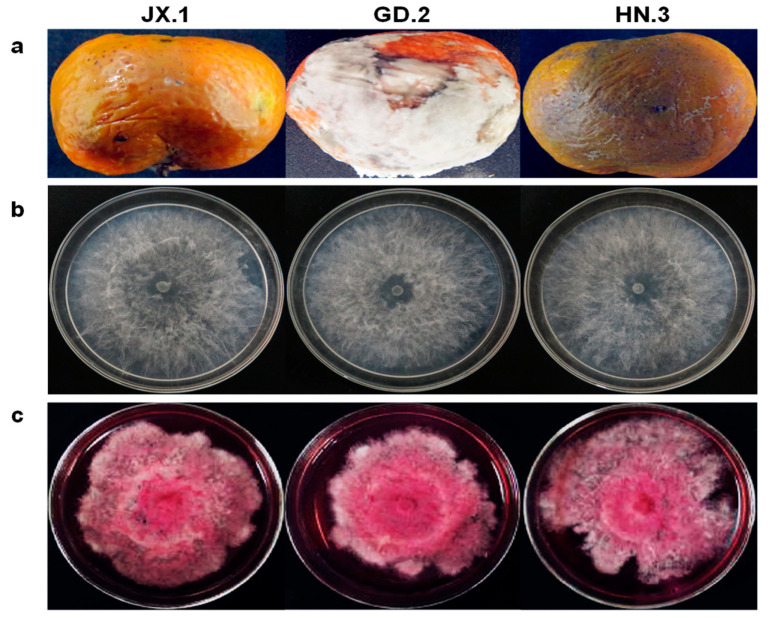
Symptoms of the diseased fruit and colony morphology on potato dextrose agar (PDA). (**a**) Symptoms of the diseased citrus fruit. (**b**) Colony morphology of the fungal strains JX.1, GD.2, and HN.3 on PDA incubated at 25 °C in the dark for 2 days; (**c**) Colony morphology of the fungal strains JX.1, GD.2, and HN.3 on PDA incubated at 35 °C in the dark for 40 days.

In 2018, a total of 2402 citrus fruit from four different citrus varieties (Satsuma mandarin (*Citrus unshiu*), Ponkan (*Citrus reticulata* Blanco cv. Ponkan), Nanfeng mandarin (*Citrus reticulata* cv. nanfengmiju), and Sugar orange (*Citrus reticulata* Blanco)) were taken from storage houses (15 °C) in seven provinces (Guangxi, Hubei, Zhejiang, Hunan, Jiangxi, Fujian, and Guangdong) and investigated. Except for fruit with typical green mold and blue mold symptoms, 402 diseased fruit samples were collected for fungal isolation. From the 402 decayed fruit, 450 fungal strains were isolated. Based on the morphological characteristics and internal transcribed spacer regions (ITS) sequences, most of them were identified as *Geotrichum* spp., *Colletotrichum* spp., *Diplodia* spp., *Alternaria* spp., etc. In addition, 16 strains belonging to the genus *Lasiodiplodia* were also isolated from 16 diseased fruits with soft, water-soaked lesions (Figure 1a, Table 1).

### 2.2. Molecular Characterization

**Figure 2:**
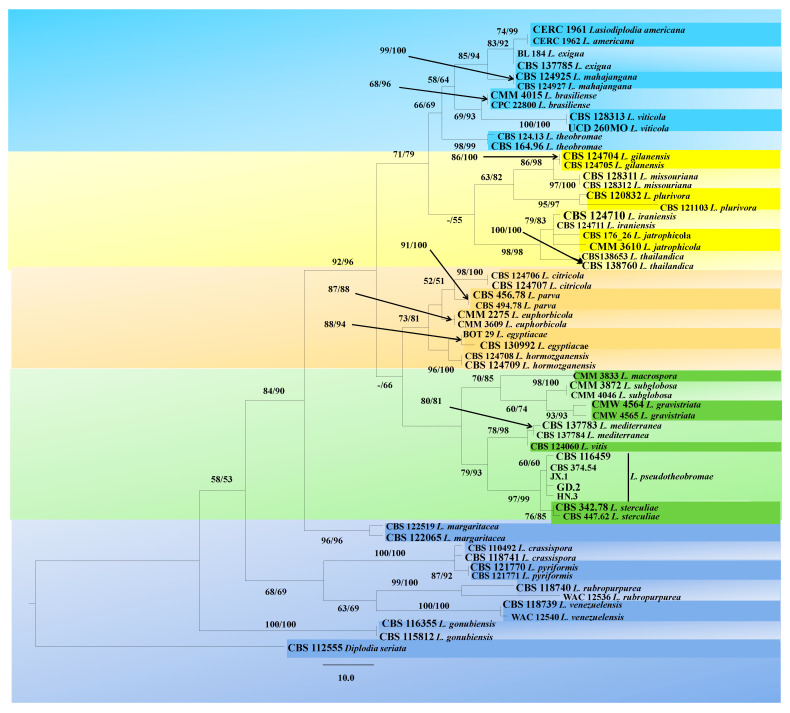
The phylogenetic tree generated from the analysis of the combined sequences of three loci, internal transcribed spacer regions (ITS), translation elongation factor 1-α gene (*TEF*), and beta-tubulin gene (*TUB*). Bootstrap support values ≥ 50% (Maximum likelihood bootstrap value (MLBS)/ Maximum parsimony bootstrap value (MPBS) are displayed at the nodes. The tree is rooted with *Diplodia seriata* (CBS 112555). Ex-type, ex-epitype, and ex-isotype cultures are indicated in bold. Isolates obtained in this study are indicated in italics. The codes of the isolates used for the phylogenetic tree are given.

Based on the multiple gene analyses, 16 strains were identified as *Lasiodiplodia pseudotheobromae*. A MegaBlast analysis showed that they exhibited 100% identity to the type strain of *L. pseudotheobromae* (CBS 116459). Since the 16 strains were of the same species, three of them from different citrus varieties and producing areas—JX.1, GD.2, and HN.3—were randomly selected for further study (Table S1). In total, 1727 characters of 62 isolates from 30 *Lasiodiplodia* species and one outgroup species (*Diplodia seriata*) were used to construct Maximum likelihood (ML) and Maximum Parsimony (MP) phylogenetic trees. The combined data included 525 characters of ITS (1–525), 550 characters of translation elongation factor 1-α gene (*TEF*) (530–1079), and 643 characters of beta-tubulin (*TUB*) (1085–1727). Based on the MP analysis, a single most parsimonious tree was constructed (tree length (TL) = 460, consistency index (CI) = 0.654, retention index (RI) = 0.854, rescaled consistency index (RC) = 0.559, homoplasy index (HI) = 0.346). Of all the characters, 175 characters were parsimony informative, 63 variable characters were parsimony uninformative, and 1,489 characters were consistent. The topology of the ML tree was similar to that of the MP tree. The isolates were grouped with *L. pseudotheobromae* but were distinguished from *L. sterculiae*, *L. vitis*, and *L. mediterranea*, the species with the closest genetic distance to *L. pseudotheobromae* (Figure 2).

### 2.3. Morphological Characteristics

**Figure 3:**
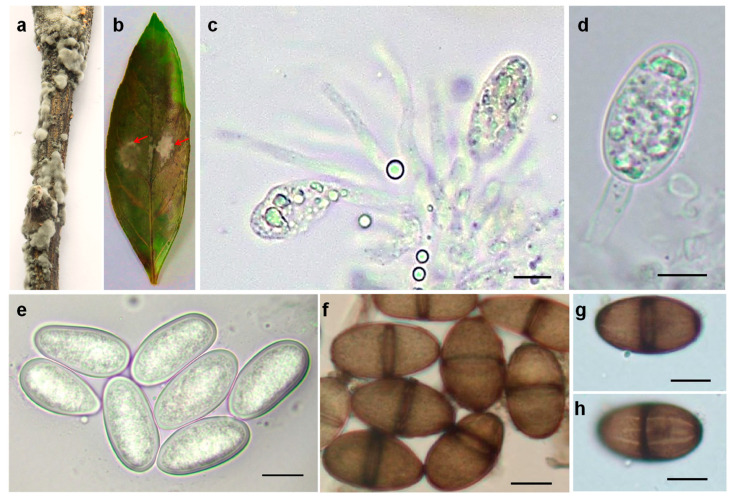
Morphology of pycnidia, conidiogenous cells, and the conidia of the strain JX.1. (**a**) Pycnidia induced on an autoclaved citrus twig. (**b**) Pycnidia induced on a citrus leaf. (**c**) Conidiogenous layer with paraphyses. (**d**) Concurrently proliferating conidiogenous cells. (**e**) Hyaline and aseptate conidia. (**f**) Septate and dark-walled conidia. (**g**,**h**) Mature conidium at two focal planes to show the longitudinal striations. Bars = 10 µm.

At first, the colonies were white with sparse, fluffy aerial mycelia. After one week, they became a pale gray with dark pigment (Figure 1b). Pycnidia were dark brown to black, solitary, globose, and intraepidermal (Figure 3a,b). The pseudparaphyses were hyaline, cylindrical-shaped, aseptate, sometimes branched, end-rounded, and arising among the conidiogenous cells (Figure 3c). Conidiogenous cells were hyaline, smooth, cylindrical, slightly swollen at the base, and holoblastic (Figure 3d). Conidia were ellipsoidal, apex and base-rounded, widest at the middle, and thick-walled. Immature conidia were colorless, hyaline, and aseptate, while mature conidia were dark brown (Figure 3e,f), one-septate with longitudinal striations (Figure 3g,h). The conidial dimensions were (22.5−) 27.8 (−32) µm × (14.5−) 17 (−20) µm ($\bar{x}$ = 27.8 × 17 µm, n = 50).

The strain JX.1 could grow between 10 and 35 °C. The optimal growth temperature was 30 °C (Figure S1). Meanwhile, all three of the strains JX.1, GD.2, and HN.3 produced a reddish pigment at 35 °C (Figure 1c).

### 2.4. Pathogenicity Assay

**Figure 4:**
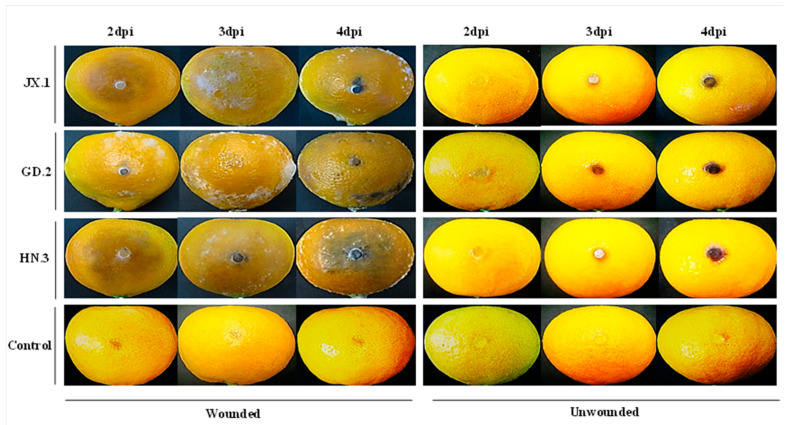
Pathogenicity of strains JX.1, GD.2, and HN.3 on Satsuma mandarin (*Citrus unshiu*) fruit. The inoculated fruit were placed in a plastic chamber maintained near 95% relative humidity, incubated at 25 °C with 12 h of light and 12 h of dark for 4 days.

In the pathogenicity assay, mature fruits of the Satsuma mandarin were used to test the virulence of the isolates. Firstly, discoloration and necrosis appeared on the fruit surface. Then, the lesions enlarged rapidly, became soft and rotten, and were covered with sparse white mycelia. The lesions spread to the whole fruit within 3 to 4 days after the occurrence of necrosis. Finally, the fruit became soggy. The onset of lesions emerged on wounded Satsuma mandarin fruit within two days of inoculation (dpi) (Figure 4). No symptoms of decay were observed on the unwounded fruit. The recovered isolates showed identical morphological traits to the original ones.

## 3. Discussion

In this study, 16 *L. pseudotheobromae* strains were isolated from rotten postharvest citrus fruit which were collected from storage rooms in seven provinces of China. This is the first report that *L. pseudotheobromae* causes fruit rot on citrus in China.

The conidiaomata of the isolates formed on citrus twigs, producing slowly maturing conidia with thick walls and longitudinal striations, which are typical morphological features of the genus *Lasiodiplodia* [4]. Furthermore, the pseudparaphyses were mostly aseptate. The colonies can grow at 10 °C and produce a dark pink pigment at 35 °C on potato dextrose agar (PDA). These morphological characteristics are consistent with those of *L. pseudotheobromae* [5,6]. Combined with the results of the phylogenetic analysis, these isolates were identified as *L. pseudotheobromae*.

Based on its morphological and molecular characteristics, *L. pseudotheobromae* has been distinguished from *L. theobromae* as a cryptic species [5]. It is a common pathogen of tropical and subtropical plants and has a wide host range, including *Acacia confusa*, *Albizia falcataria*, *Eucalyptus* sp., *Mangifera sylvaticaand*, *Paulownia fortune*, and *Vitis vinifera*, etc. [5,7,8,9,10,11,12,13,14,15,16,17,18]. *L. pseudotheobromae* was also isolated from the leaf and twig of *Magnolia candolii* as an endophyte and a saprobe [19]. It has been reported to be associated with citrus stem-end rot disease in Bangladesh and the postharvest fruit rot of lemon in Turkey [20,21].

It is difficult to evaluate the harm of *L. pseudotheobromae* to citrus fruit during the growth and storage period. Although the fungus can artificially infect citrus twigs and leaves and produce pycnidia, whether it can infect citrus in the field is still unknown. Due to the wide host range of *L. pseudotheobromae*, the primary inocula of this disease might also come from other plants in the surrounding area. At the same time, since the fungus has also been found to be an endophyte in several tropical and subtropical trees [19,22], whether it could endogenously grow in citrus trees should also be investigated. Follow-up research is needed to clarify the primary inocula and the inoculum source of the disease.

## 4. Materials and Methods 

### 4.1. Fungal Isolates

In 2018, disregarding fruit with typical green mold and blue mold symptoms, 402 diseased fruit samples of 4 different citrus varieties (Satsuma mandarin (*Citrus unshiu*), Ponkan (*Citrus reticulata* Blanco cv. Ponkan), Nanfeng mandarin (*Citrus reticulata* cv. nanfengmiju), and Sugar orange (*Citrus reticulata* Blanco) were collected from storage houses (15 °C) in seven provinces (Guangxi, Hubei, Zhejiang, Hunan, Jiangxi, Fujian, and Guangdong). Fungal isolation was performed as previously described [23]. Single-spore isolation was carried out for all the strains after conidiation.

### 4.2. Molecular Characterization

Genomic DNA was extracted using the CTAB method [24]. Fragments of ITS regions, *TEF* gene (*TEF*), and *TUB* gene were amplified by polymerase chain reaction (PCR) with primer pairs ITS1/ITS4, EF1-688F/EF1-1251R, and Bt2a/Bt2b, respectively [5,25,26]. The annealing temperatures were 54 °C for the partial ITS and 55 °C for the partial *TEF* and *TUB*, as mentioned previously. The size of the PCR products was verified by gel electrophoresis in Tris-borate-EDTA (TBE) buffer using 1% agarose gel. The PCR products were purified using E.Z.N.A.^®^ Gel Extraction Kits (Omega Bio-tek, Norcross, GA, USA) and then cloned into the vector pMD19-T (TaKaRa, Dalian, China). Sequencing was carried out at Wuhan Tianyi Huiyuan Biotechnology Co., Ltd. For each amplicon, three clones were selected for sequencing and assembly to avoid point mutations introduced by PCR and sequencing. The forward and reverse sequences were assembled by BioEdit v.7.2.5 [27]; the sequences of the three loci were deposited in GenBank (www.ncbi.nlm.nih.gov).

### 4.3. Phylogenetic Analysis

Based on a preliminary identification by MegaBlast search for ITS regions, all the available type strains with the closest genetic distance were selected for phylogenetic analysis (Table S1). The sequences of the ITS, *TEF*, and *TUB* loci were aligned by the MAFFT online service of the European Bioinformatics Institute [28] with L-INS-I iterative refinement method, and manual adjustment was performed when necessary by BioEdit v.7.2.5. Based on the combined dataset of ITS, *TEF*, and *TUB***,** the ML tree was constructed with 1000 replicates of bootstrap using RAxML-HPC BlackBox v.8.2.10 [29] on the CIPRES Science Gateway v.3.3 Web Portal (https://www.phylo.org). The MP trees were generated with 1000 replicates using Phylogenetic Analyses Using Parsimony (PAUP*) v.4.0b10 [30]. The TL, cCI, RI, RC, and HI were calculated for parsimony and the bootstrap analyses.

### 4.4. Morphological Characterization

To observe the colony morphology, all the strains were incubated on PDA at 25 °C in the dark. Meanwhile, to determine their optimal growth temperatures, the strains were incubated at 10, 15, 20, 25, 30, or 35 °C in the dark. After conidiation on autoclaved twig and leaf of a Satsuma mandarin at 25 °C with diffused daylight for 7 days, the morphology of the conidiogenous cells, paraphyses, and conidia were observed. The dimensions of 50 conidia were also measured under a microscope.

### 4.5. Pathogenicity Assay

To fulfill the Koch’s postulates, the pathogenicity of the strains was tested on the detached fruit of Satsuma mandarin. In brief, healthy, mature citrus fruit were surface-sterilized with 75% ethanol and then rinsed with sterile water. After being air dried, the fruit were wounded by an aseptic puncture. Mycelial plugs (6 mm in diameter) cut off from the margin of the activated colonies were transferred onto the wounded or unwounded citrus fruit. Plugs of PDA media were used as controls. The fruit were then incubated at 25 °C and 95% relative humidity with 12 h of light and 12 h of dark for 4 days. For each strain, 8 wounded and 8 unwounded fruit were inoculated, and the experiments were independently repeated twice. To authenticate the pathogens, the causal agents were reisolated.

## Figures and Tables

**Table 1:** Samples used in this study.

Citrus Varieties	Location	*Lasiodiplodia* Strain	No. of Rotten Fruit Except for Those with Green and Blue Mold Symptoms	Total No. of Investigated Fruit	Incidence (%)
*Citrus unshiu*	Guangxi, Guilin	1	27	225	0.44
	Hunan, Huaihua	5	76	320	1.56
	Hubei, Wuhan	1	69	258	0.39
	Hubei, Yicang	1	18	344	0.29
	Zhejiang, Taizhou	2	20	259	0.77
*Citrus reticulata* Blanco cv. Ponkan	Fujiang, Quanzhou	1	7	92	1.09
*Citrus reticulata* cv. nanfengmiju	Jiangxi, Nanfeng	3	111	566	0.53
*Citrus reticulata* Blanco	Guangdong, Quangzhou	2	74	338	0.59

## Data Availability

All sequence data are available in NCBI GenBank following the accession numbers in the manuscript.

## Supplementary Materials

The following are available online at https://www.mdpi.com/2223-7747/10/2/202/s1. Figure S1. Effects of ambient temperature on the mycelial growth of strain JX.1. The letters indicate a significant difference (*p* < 0.05). Table S1. GenBank accession numbers of isolates used in this study.
