# Supplementary material for: Identification of Lasiodiplodia pseudotheobromae Causing Fruit Rot of Citrus in China
Source: Plants (Basel). 2021 Jan 21;10(2):202. doi: 10.3390/plants10020202 (PMC7911317; doi:10.3390/plants10020202)
Supplement: Supplementary file 1 [file plants-10-00202-s001.zip › Supplementary Files/Table S1.docx]

**Table S1.** GenBank accession numbers of isolates used in this study

| **Species** | **Culture No.** | **Substrate** | | **Origin** | **Collector(s)** | **GenBank No.** | | |
| --- | --- | --- | --- | --- | --- | --- | --- | --- |
|  |  |  |  |  |  | **ITS** | ***TEF*** | ***TUB*** |
| *Lasiodiplodia americana* | **CERC 1961** = CFCC 500+B5:B715,+B5:B70 ex-type | *Pistacia vera* cv. Kerman, twigs | | USA: Arizona | T.J. Michailides | KP217059 | KP217067 | KP217075 |
| *Lasiodiplodia americana* | CERC 1962 = CFCC 50066 | *Pistacia vera* cv. Kerman, twigs | | USA: Arizona | T.J. Michailides | KP217060 | KP217068 | KP217076 |
| *Lasiodiplodia brasiliense* | **CMM 4015** = URM 7118, ex-type | *Mangifera indica*, stems | | Brazil | M.W. Marques | JX464063 | JX464049 | – |
| *Lasiodiplodia brasiliense* | CPC 22800 | *Mangifera indica*, asymptomatic twigs | | Thailand | T. Trakunyingcharoen | KJ193643 | KJ193687 | – |
| *Lasiodiplodia citricola* | CBS 124706 = IRAN 1521C | *Citrus* sp., twigs | | Iran | A. Shekari | GU945353 | GU945339 | KP872406 |
| *Lasiodiplodia citricola* | **CBS 124707** = IRAN 1522C = CJA 72, ex-type | *Citrus* sp., twigs | | Iran | J. Abdollahzadeh & A. Javadi | GU945354 | GU945340 | KP872405 |
| *Lasiodiplodia crassispora* | CBS 110492 = CPC 5047 | *Vitis vinifera*, vine | | Argentina | S. Denman | EF622086 | EF622066 | EU673134 |
| *Lasiodiplodia crassispora* | **CBS 118741** = WAC 12533 = CMW 14691, ex-type | *Santalum album* | | Australia: Western Australia | T. Burgess | DQ103550 | EU673303 | EU673133 |
| *Lasiodiplodia egyptiacae* | BOT-29 | *Mangifera indica*, leaf | | Egypt | A. Ismail | JN814401 | JN814428 | – |
| *Lasiodiplodia egyptiacae* | **CBS 130992** = BOT-10, ex-type | *Mangifera indica*, leaf | | Egypt | A. Ismail | JN814397 | JN814424 | – |
| *Lasiodiplodia euphorbicola* | **CMM 2275** = URM 7117, ex-type of L. marypalme | *Carica papaya*, fruit | | Brazil | J.H.A. Monteiro | KC484843 | KC481567 | – |
| *Lasiodiplodia euphorbicola* | **CMM 360**9, ex-type of L. euphorbicola | *Jatropha curcas*, collar and root rot | | Brazil | A.R. Machado & O.L. Pereira | KF234543 | KF226689 | KF254926 |
| *Lasiodiplodia exigua* | BL184 | *Retama raetam*, branch canker | | Tunisia | B.T. Linaldeddu | KJ638318 | KJ638337 | – |
| *Lasiodiplodia exigua* | **CBS 137785** = BL104, ex-type | *Retama raetam*, branch canker | | Tunisia | B.T. Linaldeddu | KJ638317 | KJ638336 | – |
| *Lasiodiplodia gilanensis* | **CBS 124704** = IRAN 1523C, ex-type | *Citrus* sp., fallen twigs | | Iran | J. Abdollahzadeh & A. Javadi | GU945351 | GU945342 | KP872411 |
| *Lasiodiplodia gilanensis* | CBS 124705 = IRAN 1501C | *Citrus* sp., fallen twigs | | Iran | J. Abdollahzadeh & A. Javadi | GU945352 | GU945341 | KP872412 |
| *Lasiodiplodia gonubiensis* | **CBS 115812** = CMW 14077, ex-type | *Syzygium cordatum* | | South Africa | D. Pavlic | AY639595 | DQ103566 | DQ458860 |
| *Lasiodiplodia gonubiensis* | **CBS 116355** = CMW 14078 = BOT2780, ex-paratype | *Syzigium cordatum* | | South Africa | D. Pavlic | AY639594 | DQ103567 | EU673126 |
| *Lasiodiplodia hormozganensis* | CBS 124708 = IRAN 1498C | *Mangifera indica*, twigs | | Iran | J. Abdollahzadeh & A. Javadi | GU945356 | GU945344 | KP872414 |
| *Lasiodiplodia hormozganensis* | **CBS 124709** = IRAN 1500C, ex-type | *Olea* sp., twigs | | Iran | J. Abdollahzadeh & A. Javadi | GU945355 | GU945343 | KP872413 |
| *Lasiodiplodia iraniensis* | **CBS 124710** = IRAN 1520C, ex-type | *Salvadora persica*, twigs | | Iran | J. Abdollahzadeh & A. Javadi | GU945346 | GU945334 | KP872415 |
| *Lasiodiplodia iraniensis* | CBS 124711 = IRAN 1502C | *Juglans* sp., twigs | | Iran | A. Javadi | GU945347 | GU945335 | KP872416 |
| *Lasiodiplodia jatrophicola* | CBS 176.26 | *Gossypium* sp. | | – | – | KX464136 | KX464629 | KX464900 |
| *Lasiodiplodia jatrophicola* | **CMM 3610**, ex-type | *Jatropha curcas*, collar and root rot | | Brazil | A.R. Machado & O.L. Pereira | KF234544 | KF226690 | KF254927 |
| *Lasiodiplodia macrospora* | **CMM 383**3, ex-type | *Jatropha curcas*, collar and root rot | | Brazil | A.R. Machado & O.L. Pereira | KF234557 | KF226718 | KF254941 |
| *Lasiodiplodia mahajangana* | **CBS 124925** = CMW 27801, ex-type | *Terminalia catappa*, healthy branches | | Madagascar | J. Roux | FJ900595 | FJ900641 | FJ900630 |
| *Lasiodiplodia mahajangana* | CBS 124927 = CMW 27820 | *Terminalia catappa*, healthy branches | | Madagascar | J. Roux | FJ900597 | FJ900643 | FJ900632 |
| *Lasiodiplodia margaritacea* | **CBS 122519** = CMW 26162 = MOZ 11A, ex-type | *Adansonia gibbosa* | | Australia: Western Australia | T.I. Burgess & M.J. Wingfield | EU144050 | EU144065 | KX464903 |
| *Lasiodiplodia mediterranea* | **CBS 137783** = BL1, ex-type | *Quercus ilex*, branch canker | | Italy | B.T. Linaldeddu | KJ638312 | KJ638331 | – |
| *Lasiodiplodia mediterranea* | CBS 137784 = BL101 | *Vitis vinifera*, brown stripe under the bark | | Italy | S. Serra | KJ170150 | KJ170151 | – |
| *Lasiodiplodia missouriana* | **CBS 128311** = UCD 2193MO, ex-type | Wedge-shape canker of grapevine cv.  Catawba (complex hybrid of North  America Vitis species and *Vitis vinifera*) | | USA: Missouri | K. Striegler & G.M. Leavitt | HQ288225 | HQ288267 | – |
| *Lasiodiplodia missouriana* | CBS 128312 = UCD 2199MO | Wedge-shape canker of grapevine cv.  Catawba (complex hybrid of North  America Vitis species and *Vitis vinifera*) | | USA: Missouri | K. Striegler & G.M. Leavitt | HQ288226 | HQ288268 | – |
| *Lasiodiplodia parva* | **CBS 456.78**, ex-type | Cassava-field soil | | Colombia | O. Rangel | EF622083 | EF622063 | KP872419 |
| *Lasiodiplodia parva* | CBS 494.78 | Cassava-field soil | | Colombia | O. Rangel | EF622084 | EF622064 | EU673114 |
| *Lasiodiplodia plurivora* | **CBS 120832** = CPC 5803, ex-type | *Prunus salicina*, wood canker | | South Africa | U. Damm | EF445362 | EF445395 | KP872421 |
| *Lasiodiplodia plurivora* | CBS 121103 = CPC 4583 | *Vitis vinifera* | | South Africa | F. Halleen | AY343482 | EF445396 | KX464905 |
| *Lasiodiplodia pseudotheobromae* | **CBS 116459** = KAS 2, ex-type | *Gmelina arborea* | | Costa Rica | J. Carranza- Velazquez | EF622077 | EF622057 | EU673111 |
| *Lasiodiplodia pseudotheobromae* | CBS 374.54 | | *Coffea* sp. | Zaire | – | KX464139 | KX464633 | KX464906 |
| *Lasiodiplodia pyriformis* | **CBS 121770** = CMW 25414 = CAMS 1169, ex-type | *Acacia mellifera* | | Namibia | F.J.J. van der Walt & J. Roux | EU101307 | EU101352 | – |
| *Lasiodiplodia rubropurpurea* | **CBS 118740** = WAC 12535 = CMW 14700, ex-type | *Eucalyptus grandis*, canker | | Australia | T.I. Burgess | DQ103553 | EU673304 | EU673136 |
| *Lasiodiplodia rubropurpurea* | WAC 12536 | *Eucalyptus grandis*, canker | | Australia | T.I. Burgess | DQ103554 | DQ103572 | KP872425 |
| *Lasiodiplodia sterculiae* | **CBS 342.78**, ex-type | *Sterculia oblonga* | | Germany | S. Bruhn | KX464140 | KX464634 | KX464908 |
| *Lasiodiplodia sterculiae* | CBS 447.62 | *Citrus aurantium*, fruit | | Suriname | – | EF622081 | EF622060 | EU673112 |
| *Lasiodiplodia subglobosa* | **CMM 3872**, ex-type | *Jatropha curcas*, collar and root rot | | Brazil | A.R. Machado & O.L. Pereira | KF234558 | KF226721 | KF254942 |
| *Lasiodiplodia subglobosa* | CMM 4046 | *Jatropha curcas* | | Brazil | A.R. Machado & O.L. Pereira | KF234560 | KF226723 | KF254944 |
| *Lasiodiplodia thailandica* | CBS 138653 = CPC 22755 | *Phyllanthus acidus*, petiole | | Thailand | T. Trakunyingcharoen | KM006433 | KM006464 | – |
| *Lasiodiplodia thailandica* | **CBS 138760** = CPC 22795, ex-type | *Mangifera indica*, twigs | | Thailand | T. Trakunyingcharoen | KJ193637 | KJ193681 | – |
| *Lasiodiplodia venezuelensis* | **CBS 118739** = WAC 12539 = CMW 13511, ex-type | *Acacia mangium*, wood | | Venezuela | S. Mohali | DQ103547 | EU673305 | EU673129 |
| *Lasiodiplodia venezuelensis* | WAC 12540 | *Acacia mangium*, wood | | Venezuela | S. Mohali | DQ103548 | DQ103569 | KP872427 |
| *Lasiodiplodia viticola* | **CBS 128313** = UCD 2553AR, ex-type | Wedge-shape canker of grapevine cv.  Vignoles (complex hybrid of North  America Vitis species and *Vitis vinifera*) | | USA: Arkansas | R.D. Cartwright & W.D. Gubler | HQ288227 | HQ288269 | HQ288306 |
| *Lasiodiplodia vitis* | CBS 124060 = PvPa, ex-type | *Vitis vinifera*, wood fragment | | Italy | – | KX464148 | KX464642 | KX464917 |
| *Lasiodiplodia gravistriata* | **CMW 4564**, ex-type | Anacardium | | Brazil | M.S.B.Netto | KT250949 | KT250950 | – |
| *Lasiodiplodia gravistriata* | CMW 4565 | Anacardium | | Brazil | M.S.B.Netto | KT250947 | KT266812 | – |
| *Lasiodiplodia pyriformis* | **CMW 25415**=CBS121771 , ex-type | *Acacia mellifera* | | Namibia | F.J.J. van der Walt & J. Roux | EU101308 | EU101353 | – |
| *Lasiodiplodia viticola* | **UCD 2604MO**, ex-type | Wedge-shape canker of grapevine cv.  Vignoles (complex hybrid of North  America Vitis species and *Vitis vinifera*) | | USA: Arkansas | K.Striegler & W.D. Gubler | HQ288228 | HQ288270 | HQ288307 |
| *Lasiodiplodia margaritacea* | **CBS 122065**, ex-type | *Adansonia gibbosa* | | Australia: Western Australia | – | EU144051 | – | – |
| *Diplodia seriata* | **CBS 112555** = HAP 052 = CAP 063, ex-epitype | *Vitis vinifera*, dead stems | | Portugal | A.J.L. Phillips | AY259094 | AY573220 | DQ458856 |
| *Lasiodiplodia pseudotheobromae* | JX.1 | *Citrus reticulata* cv. nanfengmiju, fruit | | China | J.H.Chen & Y.LIN | MN701082 | MT4778657 | MT478660 |
| *Lasiodiplodia pseudotheobromae* | GD.2 | *Citrus reticulata* Blanco, fruit | | China | J.H.Chen & Y.LIN | MN701198 | MT4778658 | MT478661 |
| *Lasiodiplodia pseudotheobromae* | HN.3 | *Citrus unshiu*, fruit | | China | J.H.Chen & Y.LIN | MN701063 | MT4778659 | MT478662 |

**AR:** Personal collection of A. Rossman**; ATCC:** American Type Culture Collection, Virginia, USA; BL: Personal number of B.T. Linaldeddu; Bot: Personal number of S. Denman; **BRIP:** Culture collection, Queensland Department of Agriculture and Fisheries, Queensland, Australia; **CAP:** Personal culture of A.J.L. Phillips; **CBS:** CBS-KNAW Fungal Biodiversity Centre, Utrecht, The Netherlands; **CCTCC:** China Center for Type Culture Collection, Wuhan University, China; **CFCC:** China Forestry Culture Collection Center, Beijing, China; **CMM:** Culture Collection of Phytopathogenic Fungi “Prof. Maria Menezes”, Universidade Federal Rural de Pernambuco, Recife, Brazil; **CMW:** Tree Pathology Co-operative Program, Forestry and Agricultural Biotechnology Institute, University of Pretoria, South Africa; **CPC:** Working collection of P.W. Crous, housed at CBS; **DAR:** Plant Pathology Herbarium, Orange Agricultural Institute, Forest Road, Orange. NSW 2800, Australia; **DSM:** Deutsche Sammlung von Mikrorrganismen und Zellkulturen GmbH, Braunschweig, Germany; **ETH:** Swiss Federal Institute of Technology Culture Collection, Zurich, Switzerland; **ICMP:** International Collection of Microorganisms from Plants, Landcare Research, Aukland, New Zealand; **IFO:** Institute for Fermentation, Osaka, Japan; **IMI:** International Mycological Institute, CBI-Bioscience, Egham, Bakeham Lane, UK; **IRAN:** Iranian Fungal Culture Collection, Iranian Research Institute of Plant Protection, Iran; **JL:** Personal culture collection of J. Luque, IRTA, Barcelona, Spain; **JT:** Personal number of J.E. Taylor; **ME:** Personal number of M. Elliott; **MFLUCC:** Mae Fah Luang University Culture Collection, Chiang Rai, Thailand; **MUCC** (Australia): Murdoch University Culture Collection, Murdoch, Australia; SL: Personal number of S. Marincowitz; UAMH: University of Alberta Microfungus Collection and Herbarium, Edmonton, Alberta, Canada; **UCROK:** Culture collection, University of Riverside, California, USA; **UPSC:** Uppsala University Culture Collection of Fungi, Botanical Museum University of Uppsala, Uppsala, Sweden; **VPRI:** Victorian Department of Primary Industries, Knoxfield, Australia; **WAC:** Department of Agriculture, Western Australia Plant Pathogen Collection, South Perth, Western Australia.

**ITS**: internal transcribed spacer regions and intervening 5.8S rRNA gene; **tef1**: partial translation elongation factor 1-alpha gene; **tub2**: partial beta-tubulin gene.
