# Supplementary figures and images for: Identification of Lasiodiplodia pseudotheobromae Causing Fruit Rot of Citrus in China
Source: Plants (Basel). 2021 Jan 21;10(2):202. doi: 10.3390/plants10020202 (PMC7911317; doi:10.3390/plants10020202)

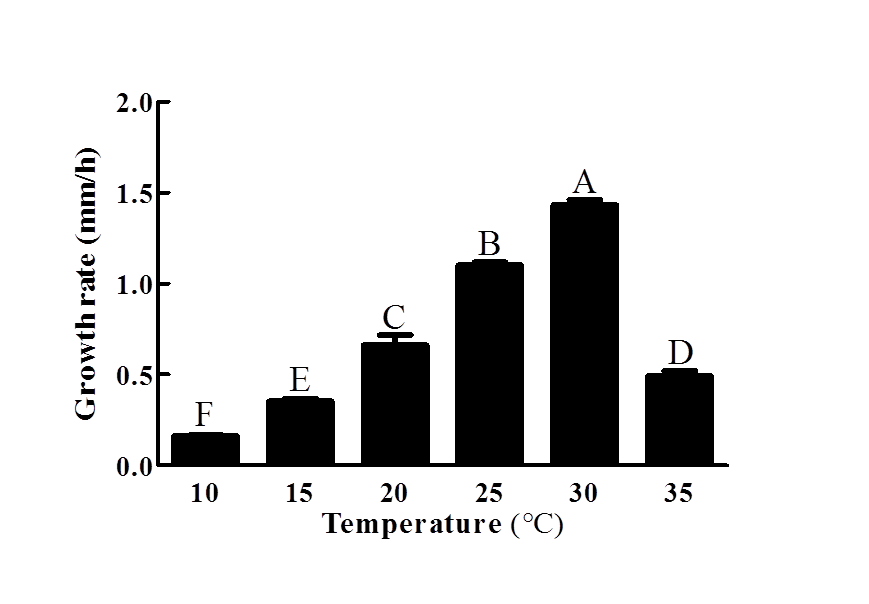

Supplement: Supplementary file 1 [file plants-10-00202-s001.zip › Supplementary Files/FigS1.tif]
